# Big data in corneal diseases and cataract: Current applications and future directions

**DOI:** 10.3389/fdata.2023.1017420

**Published:** 2023-02-01

**Authors:** Darren S. J. Ting, Rashmi Deshmukh, Daniel S. W. Ting, Marcus Ang

**Affiliations:** ^1^Academic Unit of Ophthalmology, Institute of Inflammation and Ageing, University of Birmingham, Birmingham, United Kingdom; ^2^Birmingham and Midland Eye Centre, Birmingham, United Kingdom; ^3^Academic Ophthalmology, School of Medicine, University of Nottingham, Nottingham, United Kingdom; ^4^Department of Cornea and Refractive Surgery, LV Prasad Eye Institute, Hyderabad, India; ^5^Singapore National Eye Centre, Singapore Eye Research Institute, Singapore, Singapore; ^6^Department of Ophthalmology and Visual Sciences, Duke-National University of Singapore (NUS) Medical School, Singapore, Singapore

**Keywords:** big data, cornea, cataract, clinical registry, artificial intelligence, electronic health record (EHR), mHealth, ophthalmology (MeSH)

## Abstract

The accelerated growth in electronic health records (EHR), Internet-of-Things, mHealth, telemedicine, and artificial intelligence (AI) in the recent years have significantly fuelled the interest and development in big data research. Big data refer to complex datasets that are characterized by the attributes of “5 Vs”—variety, volume, velocity, veracity, and value. Big data analytics research has so far benefitted many fields of medicine, including ophthalmology. The availability of these big data not only allow for comprehensive and timely examinations of the epidemiology, trends, characteristics, outcomes, and prognostic factors of many diseases, but also enable the development of highly accurate AI algorithms in diagnosing a wide range of medical diseases as well as discovering new patterns or associations of diseases that are previously unknown to clinicians and researchers. Within the field of ophthalmology, there is a rapidly expanding pool of large clinical registries, epidemiological studies, omics studies, and biobanks through which big data can be accessed. National corneal transplant registries, genome-wide association studies, national cataract databases, and large ophthalmology-related EHR-based registries (e.g., AAO IRIS Registry) are some of the key resources. In this review, we aim to provide a succinct overview of the availability and clinical applicability of big data in ophthalmology, particularly from the perspective of corneal diseases and cataract, the synergistic potential of big data, AI technologies, internet of things, mHealth, and wearable smart devices, and the potential barriers for realizing the clinical and research potential of big data in this field.

## 1. Introduction

The concept of big data was first introduced in 1990s to capture dataset that are too complex to be stored and analyzed using traditional computer software (Mallappallil et al., 2020). It was previously defined as data that display the characteristics of “3 Vs”—volume, velocity and variety (Mooney et al., 2015). Additional attributes such as veracity and value have also been suggested to fully capture the true nature and values of big data (known as the “5 Vs”).^1^

In the recent years, the accelerated growth in electronic health records (EHR), disease registries, biobanks, mHealth, Internet-of-Things (IoT), telemedicine, and artificial intelligence (AI) have helped unlock the multi-faceted potential of big data (Chiang et al., 2018; Li et al., 2021; Sahu et al., 2021). Compared to traditional dataset, the wealth of information provided by big data (which are often derived from large-scale or nationwide studies) can facilitate comprehensive and timely examination of the epidemiology, trends, characteristics, outcomes, and prognostic factors of the diseases (Roski et al., 2014; Mallappallil et al., 2020). In addition, the findings help to guide public health policies in terms of risk factors modulation, disease prevention and control, optimization of healthcare service provision, and allocation of research funding targeting more prevalent diseases (Roski et al., 2014).

**Figure 1:**
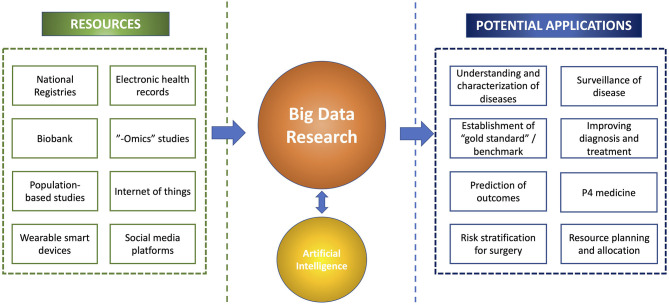
An overview of the big data research, including the resources, potential synergism with artificial intelligence, and clinical applications.

The multi-dimensional values of big data have been increasingly capitalized in many branches of medicine, including ophthalmology (Cheng et al., 2020; Li et al., 2021). One of the best examples relates to the recent use of big data in understanding the trends and spread of COVID-19, risk factors, treatment outcomes, and morbidity/mortality, which helped inform the clinical practice and public health policies (Haleem et al., 2020; Ting et al., 2020c; Villanustre et al., 2021). Furthermore, big data have enabled the development of highly accurate AI algorithms (which are often data-hungry) in diagnosing a wide range of medical diseases as well as discovering new patterns or associations of diseases that are previously unknown to us (Figure 1) (Ting D. S. W. et al., 2017; Milea et al., 2020; Mehta et al., 2021; Rim et al., 2021; Ting et al., 2021b).

Within the field of ophthalmology, there is a rapidly expanding pool of large clinical registries, epidemiological studies, omics studies, and biobanks through which big data can be accessed (Chua et al., 2019; Tan et al., 2019). In view of the increased availability and accessibility of big data and recent technological advancements, this paper aimed to provide a succinct overview of the availability, clinical applicability and future potentials of big data in ophthalmology, particularly from the perspective of corneal diseases and cataract.

## 2. Big data in corneal diseases

According to a recent report by the World Health Organization (WHO), corneal opacity represents the 5th leading cause of blindness globally (Flaxman et al., 2017).^2^ It is also estimated that ~6 million people suffer from moderate to severe visual impairment secondary to corneal opacity, including non-trachomatous and trachomatous-related cases (see text footnote 2). More importantly, corneal blindness has been shown to be significantly more prevalent in low- and middle-income countries (LMICs), mainly due to limited healthcare resources, higher rate of ocular trauma, poor environmental and personal hygiene, malnutrition, and lower educational level, amongst others (Flaxman et al., 2017; Porth et al., 2019; Ting et al., 2021e). Given the enormity of corneal blindness globally and the significant mismatch between the disease burden and the availability of healthcare resources and workforce, strategic measures are urgently needed.

Within the field of cornea, there is an increasing pool of large corneal registries and epidemiological studies that contains rich resources of big data. These include corneal transplant registries, infectious keratitis studies, genomic studies, large ophthalmology-related registries, EMR-based platforms, and biobanks (Keenan et al., 2012; Chiang et al., 2018; Donthineni et al., 2019). These cornea-related big data enable a better grasp of the prevalence, risk factors, outcomes, and impact of various corneal diseases, which in turn allow for more effective formulations of various therapeutic and preventative strategies in tackling the diseases. In this section, we summarize the main cornea-related big data in various countries and their impact on clinical practice, research and public policies.

### 2.1. Corneal transplant registries

Corneal transplantation or keratoplasty is the most common type of transplantation performed worldwide (Tan et al., 2012). Currently it remains the main method for restoring corneal clarity and vision in patients with visually debilitating corneal diseases (Tan et al., 2012). However, the persistent shortage of donor corneas has posed significant barriers to successful corneal transplantations (Gain et al., 2016). This has also led to the implementation of various innovative measures, with an aim to improve the eye donation rate (Rithalia et al., 2009; Ting et al., 2016a), utilization of donor corneas (Ting et al., 2016b; Gupta et al., 2018), and reduction of the need for donor corneas (Kinoshita et al., 2018; Ting et al., 2022). In order to tackle this persistent barrier, a wide range of national corneal graft registries and eye banks have been established across the world, including the US, the UK, Europe, India, Australia, and Singapore, amongst others (Table 1) (Tan et al., 2015, 2019; Sharma et al., 2019; Dunker et al., 2021).^3^

**Table 1:** Summary of main corneal transplant registries and institutions in the world, categorized by continents.

**Countries**	**Corneal transplant registries (and institutions)**
**Multi-continent**	
Global	Global Alliance of Eye Bank Associations (GAEBA) Pan American Association of Eye Banks
**Asia**	
China	Beijing Tongren Eye Center Shandong Eye Institute
Hong Kong	Lions Eye Bank of Hong Kong
Japan	Cornea Centre and Eye Bank, Tokyo Dental College Kyoto Prefectural University of Medicine
India	Eye Bank Association of India (EBAI)
Malaysia	National Transplant Registry of Malaysia
Philippines	Santa Lucia International Eye Bank of Manila
Russia	S. N. Fyodorov Eye Microsurgery State Institution
Saudi Arabia	King Khaled Eye Specialist Hospital
Singapore	Singapore Corneal Transplant Study, Singapore Eye Bank
South Korea	Korean Network for Organ Sharing (KONOS) Seoul St. Mary's Eye Hospital
Taiwan	National Taiwan University Hospital
**North and South America**	
Brazil	Brazilian Association of Organ Transplantation (ABTO)
US	Eye Bank Association of America (EBAA)
**UK and Europe**	
Europe	European Cornea and Cell Transplantation Registry
France	Centre Francois Xavier Michelet, CHU de Bordeaux, Site Pellegrin
Germany	German Ophthalmological Society (GOS)
Italy	Societa Italian Traplanto Di Cornea (S.I.TRA.C) Veneto Eye Bank Foundation
Netherland	Netherlands Institute for Innovative Ocular Surgery
Sweden	Swedish Registry for Corneal Transplant
UK	UK National Health Service (NHS) Blood and Transplant
**Australasia**	
Australia	Australian Cornea Graft Registry (ACGR)
New Zealand	New Zealand National Eye Centre
**Africa**	
Ethopia	Addis Ababa University
South Africa	Pretoria Eye Institute

Adapted from the thesis published by Tan et al. (2015).

The purposes of these national registries and eye banks are manifold. Firstly, it helps standardize the corneal donation-to-transplantation pathway nationally and identify any potential limiting factors, enabling more effective interventions to improve the conversion rate of eye donation and the utilization rate of the donated corneas (Gogia et al., 2015; Ting et al., 2016b; Sharma et al., 2019). Secondly, the prospective database can facilitate examination of the ongoing availability of donor corneas to allow for equal and fair distribution of the donor corneas across the country (Ting et al., 2016b; Gupta et al., 2018) (see text footnote 3).^4^ It also helps inform the policymakers and relevant stakeholders on the need for importation (or exportation) depending on the local supply of donor corneas. Thirdly, they provide up-to-date examination of the trends in the types and indications of keratoplasty (Keenan et al., 2012; Park et al., 2015). For instance, various studies have demonstrated a paradigm shift from penetrating keratoplasty (PK) to lamellar keratoplasty [including anterior lamellar keratoplasty (ALK) and endothelial keratoplasty (EK)] over the past decade in many countries. A recent European Cornea and Cell Transplantation Registry study of 10 centers from the Europe, the UK and Switzerland (*n* = 12913 keratoplasty) demonstrated that Descemet stripping automated endothelial keratoplasty (DSAEK) was the most commonly performed technique (46%), followed by PK (30%) and Descemet membrane endothelial keratoplasty (9%) (Dunker et al., 2021). In addition, the study demonstrated that Fuchs endothelial corneal dystrophy (FECD), regraft, pseudophakic bullous keratopathy (PBK), and keratoconus were the main indications for keratoplasty. These common indications were consistently reported in many other national studies conducted in other countries (Keenan et al., 2012; Ting et al., 2012; Park et al., 2015; Tan et al., 2015; Ang et al., 2016c; Fuest et al., 2017). Understanding of the common indications for keratoplasty provides the clinicians and researchers with a clearer picture of how the limited resources (i.e., donor corneas) are being utilized. In addition, more targeted research effort can be channeled toward these higher prevalent corneal diseases to search for alternative therapeutic strategies and reduce the need for donor corneas.

Furthermore, useful information can be obtained from these national corneal transplant registries to understand the risk factors and prognostic factors of keratoplasty, ultimately improving the clinical outcomes (Ang et al., 2011, 2012a, 2016a; Bose et al., 2013). Ang et al. (2016b) observed that patients with FECD and bullous keratopathy achieved a better long-term graft survival following Descemet membrane endothelial keratoplasty (DMEK) when compared to Descemet stripping automated endothelial keratoplasty (DSAEK) and PK. On the other hand, an Australian national study of >15,000 cases of keratoplasty demonstrated that the survival of lamellar keratoplasty (i.e., DALK and EK) fared worse than PK, with some evidence showing the influence of learning curve on the outcome of EK (Coster et al., 2014). Important prognostic factors for graft survival rate, including the indication for graft, number of previous grafts, history of ocular surface inflammation or glaucoma, corneal neovascularization, and postoperative events such as graft rejection or infection, were also identified *via* these national corneal transplantation studies (Williams et al., 2008; Ang et al., 2012b, 2014, 2020; Sibley et al., 2020). Indications such as PBK and infectious keratitis (IK) have been shown to be associated with a worse outcome compared to “low-risk” conditions such as keratoconus and FECD following keratoplasty (Tan et al., 2012), highlighting the need for improvement in the treatment strategy for certain indications (Ang and Sng, 2018).

More importantly, the registries enable examination and monitoring for any significant postoperative adverse events such as infection and endophthalmitis (Chen et al., 2015; Gauthier et al., 2017; Song et al., 2021). Edelstein et al. (2016) previously conducted a study of 354,390 keratoplasty based on the data from the Eye Bank Association of America, analyzing all adverse events following all types of keratoplasty. They observed a higher rate of fungal infection in their study compared to non-US studies and postulated that this might be due to the lack of antifungal agent used in the corneal storage medium in the US (Chen et al., 2015; Edelstein et al., 2016). It was also found that fungal keratitis and endophthalmitis were more common following EK (1.5–3 times higher risk) than PK and ALK, potentially attributed to the increased warming time associated with the preparation of EK tissues in the eye bank (Edelstein et al., 2016). These findings will allow for the refinement of the eye bank protocol in terms of processing and storage of donor corneas, ultimately leading to improved clinical outcome and safety.

### 2.2. Infectious keratitis databases

Corneal opacity is the 5th leading cause of blindness globally, with IK being the main culprit. IK was previously recognized as a “silent epidemic”, and recently, a “neglected tropical disease” status was proposed (Ung et al., 2019). The incidence is estimated to range between 2.5–799 per 100,000 population per year (Ting et al., 2021e). It can be caused by a wide range of organisms, including bacteria, fungi, viruses, and parasites, and polymicrobial infection (Ting et al., 2019b, 2021d; Khoo et al., 2020). In view of its significant impact on human health, healthcare systems and economy, it is therefore not surprising to observe a vast amount of literature on IK, encompassing the epidemiology, risk factors, clinical characteristics, causative organisms, management, and outcomes of the disease. Large IK studies published in the recent years are summarized in Table 2 (Lin et al., 2017, 2019; Tan et al., 2017; Khor et al., 2018; Peng et al., 2018; Green et al., 2019; Tavassoli et al., 2019; Asbell et al., 2020; Khoo et al., 2020; Kowalski et al., 2020; Somerville et al., 2021; Ting et al., 2021d).

**Table 2:** Summary of large-scale infectious keratitis study (>1,000 cases) in the world published between 2016 and 2021, in chronological order.

**Authors (Year)**	**Study period**	**Region**	**No. of scrapes**	**Culture positivity (%)**	**Bacteria (%)**	**Fungi (%)**	**Acanthamoeba (%)**
Somerville et al. (2021)	2014–2020	UK	3,099	47.2	51.4	0.8	0.2
Ting et al. (2021d)	2007–2019	UK	1,333	37.7	92.8	3.0	4.2
Asbell et al. (2020)^*^	2009–2018	US	6,091	100.0	100.0	–	–
Khoo et al. (2020)	2012–2016	Australia	1,052	48	64	2.3	–
Lin et al. (2019)	2010–2018	China	7,229	42.8	52.7	57.6	–
Tavassoli et al. (2019)	2006–2017	UK	2,614	38.1	91.6	6.9	1.4
Green et al. (2019)^*^	2005–2015	Australia	3,182	100.0	93.1	6.3	0.5
Kowalski et al. (2020)^*^^#^	1993–2018	US	1,387	100.0	72.1	6.7	5.2
Peng et al. (2018)	1996–2015	US	2,203	23.7	100.0	–	–
Khor et al. (2018)	2012–2014	Asia	6,563	43.1	38	32.7	2.26
Tan et al. (2017)	2004–2015	UK	4,229	32.6	90.6	7.1	2.3
Lin et al. (2017)	2009–2013	China	2,973	46.1	41.9	44.6	13.6

^*^These studies only included culture-proven IK cases.

^#^This study also included viral keratitis cases.

The clinical and laboratory data captured by these large-scale IK studies enables a better grasp of the microbiological profiles, risk factors, disease impact, and treatment response. So far, from the epidemiological standpoint, these studies have helped unveil the considerable geographical, temporal and seasonal variations in IK, which provide useful guidance to the choice of antimicrobial treatment. For example, *Staphylococci* spp. and *Pseudomonas* spp. were shown to be the most common organisms in the UK, the US and Australia (Tan et al., 2017; Tavassoli et al., 2019; Khoo et al., 2020; Kowalski et al., 2020; Ting et al., 2021d). In addition, several studies (Tan et al., 2017; Ting et al., 2018, 2021d) have identified an increasing trend of moraxella keratitis in the UK over the past decade. In contrast, a recent Asia Cornea Society Infectious Keratitis Study (ACSIKS) of more than 6,000 patients showed that fungal and bacterial infections were the main causes of IK in developing and developed countries, respectively (Khor et al., 2018). More importantly. the study observed ~50% of the eyes developed moderate visual loss (< 6/18 vision), with 46% of the performed therapeutic keratoplasty failed by 6 months' follow-up, highlighting the significant impact on the affected patients.

Studies have shown that the initial severity of the ulcer and presenting visual acuity serve as important prognostic factors for IK (Khoo et al., 2020; Ting et al., 2021a,c). Therefore understanding the risk factors *via* big data research allows for effective implantation of various preventative strategies in reducing the incidence of IK. Contact lens (CL) has been consistently identified as one of the most common risk factors for IK (Cariello et al., 2011; Keay et al., 2011; Ting et al., 2021a). In particular, the risk of CL-related IK was shown to be associated with use of expired CL and overnight CL wear (Sauer et al., 2020). Understanding of these underlying factors allow for better education among the patients and CL wearers. Trauma serves as another important risk factor for IK, particularly in the developing countries (Ganguly et al., 2011; Kaliamurthy et al., 2013). In addition, based on a population-based, cross-section sectional study, [Cornea Opacity Rural Epidemiological (CORE) study] (Gupta et al., 2017), it was found that the use of traditional eye medicine and self-medication was prevalent in the rural regions of India, which could lead to delay in seeking appropriate medical care and exacerbation of corneal diseases and/or infection. These epidemiological studies have helped improve the public awareness and call for new regulatory legislations to address these issues.

Broad-spectrum topical antimicrobial therapy serves as the current mainstay of treatment for IK, though their efficacy is being challenged by the emergence of AMR, observed in several large-scale IK studies (Lalitha et al., 2017; Asbell et al., 2020; Ting et al., 2021e). Clinically, AMR-related pathogens has been shown to negatively affect the outcome and healing time of IK (Kaye et al., 2010). In the Antibiotic Resistance Among Ocular Microorganisms (ARMOR) with data from >6,000 ocular isolates, Asbell et al. (2020) observed that ~40% of the *Staphylococci* spp. were methicillin-resistant, and many of them were multidrug resistant. On the other hand, various studies in the UK demonstrated a low rate of AMR (< 5–10%) against the commonly employed antibiotic regimens used for IK, including fluoroquinolone, cephalosporin and aminoglycoside (Tan et al., 2017; Tavassoli et al., 2019; Ting et al., 2021d). These findings emphasize the wide geographical and temporal variations in AMR for IK and the importance of updated examination in specific regions. Better knowledge of the AMR pattern could also help guide the most appropriate initial treatment for IK in each region.

### 2.3. Corneal genomic studies

The increase in large-scale genetic studies, particularly genome-wide association study (GWAS) and genome-wide linkage study (GWLS), has significantly advanced our understanding of many diseases, including corneal diseases, and offer potential novel targets for gene therapy (Riazuddin et al., 2009, 2010, 2013; Baratz et al., 2010; Burdon et al., 2011; Bykhovskaya et al., 2012; Czugala et al., 2012; Li et al., 2013; Lu et al., 2013; Sahebjada et al., 2013; Dudakova et al., 2015; Afshari et al., 2017; McComish et al., 2019). GWAS is an invaluable methodology designed to analyze common genetic variations across the whole genome, particularly single nucleotide polymorphisms (SNPs), by analyzing the genotype-phenotype associations of a disease in case-control cohorts with a large number of individuals. On the other hand, GWLS is a useful tool used to genotype a particular disease by examining families with affected and unaffected individuals (Karolak and Gajecka, 2017; Tam et al., 2019).

FECD and keratoconus are by far the two most commonly investigated corneal diseases. The research focus on these two conditions is primarily driven by the high burden and prevalence of the diseases. Moreover, they represent the most common indications for keratoplasty in many countries (Ting et al., 2012; Park et al., 2015), placing significant burden on the donor corneas. Over the years, GWAS has increasingly been used to identify genetic susceptibility regions in FECD and keratoconus (Iliff et al., 2012; Karolak and Gajecka, 2017). For instance, Hardcastle et al. (2021) recently conducted a multi-ethnic GWAS of keratoconus, including >100,000 individuals, and identified 36 significant genomic loci that were associated with keratoconus. McComish et al. (2019) discovered a novel genetic locus in PNPLA2 at chromosome 11 for keratoconus based on over 6 million genetic variants. Several novel genetic loci for FECD, including *TCF4, LAMC1* rs3768617, *LINC00970/ATP1B1* rs1200114, and *KANK4* rs7974289*5*, have also been identified (Afshari et al., 2017). GWLS have also facilitated the identification of a number of important genetic mutations linked to keratoconus, including *TGFBI, TCEB1, CAST, COL8A1*, and *LOX* genes (Karolak and Gajecka, 2017). Next-generation sequencing, which enables extensive and deep sequencing of the DNA (Londin et al., 2013), has recently been employed to detect novel mutations associated with many other types of corneal dystrophy (Zhang et al., 2019).

In view of the rapid proliferation of the genomic studies, many genetic banks, databases and web-based resources such as https://www.ncbi.nlm.nih.gov/gtr/ and https://www.omim.org/ have been created to capture and summarize the genomic association of a wide array of human diseases, including ocular diseases. The availability of these results not only help reduce unnecessary duplication of any previously conducted research, which often involves extensive time, effort and funding, but also expedite the discovery and development of new therapeutic targets *via* knowledge- and data-sharing.

### 2.4. Electronic health record-based registries and biobanks

The rapid emergence of EHR in healthcare systems in the recent years has allowed the capture and analysis of big data by the clinicians, researchers and relevant stakeholders (DesRoches et al., 2008; Day et al., 2015; Evans, 2016). One of the most notable examples in the field of ophthalmology is the Intelligent Research in Sight (IRIS). Registry, which is a US-based ophthalmic EHR registry established by the American Academy of Ophthalmology (Parke Ii et al., 2017; Chiang et al., 2018). In 2016, the IRIS Registry had already captured data from >17 million eye patients, including over a million of patients with dry eye disease (DED), and >35 million ophthalmic visits from 7,200 US-based ophthalmologists, providing valuable information on prevalence, demographic factors, risk factors, management and outcome of a wide range of ocular diseases (Chiang et al., 2018). So far, the IRIS Registry has enabled research in many fields of ophthalmology, including cornea (Anchouche et al., 2021), cataract (Pershing et al., 2020; Owen et al., 2021; Lacy et al., 2022), glaucoma (Chang et al., 2021; Olivier et al., 2021), and retina (Malhotra et al., 2021; Khanani et al., 2022). For instance, Anchouche et al. (2021) included >60,000 patients of chemical and thermal ocular injuries in the US (using the IRIS Registry data) and demonstrated a significant increase in the incidence by 20% from 2013 to 2017 in the US. In a similar vein, Donthineni et al. (2019) demonstrated the value of utilizing EHR-derived big data to estimate the incidence of DED in India as well as the predisposing factors such as age, gender, socio-economic status, and profession. Using the same EHR database of >2 million patients, Das and Basu (2022) were able to identify >20,000 patients who presented with epidemic keratoconjunctivitis and characterized the clinical features and outcomes, enabling more accurate and timely diagnosis and treatment.

Furthermore, there are nationwide databases such as the UK Biobank which also contain extensive clinical, imaging and genomic data related to the eye, including the cornea (Chua et al., 2019). UK Biobank is a large-scale biomedical database and research resource, which contains extensive health, genetic and bioimaging information from >500,000 people in the UK, with regularly update on additional follow-up data.^5^ Corneal hysteresis serves as an important biomechanical property of cornea, and it has been shown to influence the measurement of intraocular pressure and risk of glaucoma (Deol et al., 2015). Based on the data of >90,000 participants obtained from the UK Biobank, significant associations between corneal hysteresis and various demographic factors such as age, sex, and ethnicity were detected (Zhang et al., 2019). Khawaja et al. (2019) similarly identified five novel loci that are associated with corneal biomechanical properties, including corneal hysteresis and corneal resistance, which may have important implication on the pathogenesis of keratoconus. In addition, GWAS based on the UK Biobank data enabled the discovery of four novel genetic loci, including HERC2, LINC00340, NPLOC4, and ZC3H11B genes, for corneal astigmatism (Shah and Guggenheim, 2018).

## 3. Big data in cataract

Cataract is the leading cause of blindness and visual impairment globally, affecting around 94 million of the world population, particularly in the low- and middle-income countries (LMICs) (see text footnote 2) (Flaxman et al., 2017). Currently, ~20 million cases of cataract surgery are being performed each year (Wang et al., 2016), making it the most commonly performed surgery worldwide. In view of the continuous advancement in the phacoemulsification technology, surgical techniques, biometry calculation for IOL power, and IOL technology, the demand for perfect vision and no/minimal risk of surgical complication continues to rise (Erie, 2014; Ting D. S. J. et al., 2017; Sudhir et al., 2019; Day et al., 2020; Ting et al., 2020a). Furthermore, as cataract surgery is the most commonly performed ophthalmic surgery, it often used as the benchmark for assessing an ophthalmologist's surgical competence, especially during the specialist training or residency program.

To date, many national cataract databases have been established across the world (Table 3). One of the primary aims of these databases is to examine and audit the outcomes of the cataract surgery performed by the surgeons. Secondly, it also helps provide a benchmark for the visual outcome and safety of the surgery for all the cataract surgeons, with adjustment of the experience and complexity of the case-mix. In addition, these big data may also identify important factors that can predict the risk of intraoperative and postoperative complications, including posterior capsular rupture (PCR), retinal detachment, cystoid macular edema (CMO), endophthalmitis, and many others.

**Table 3:** Summary of main cataract registries in the world, categorized by continents.

**Countries**	**Cataract registries (and institutions)**
**Asia**	
China	Shanghai Cataract Operations Database
India	Aravind Eye Hospitals Registry
Israel	Israel Cataract Registry
Malaysia	Malaysia Cataract Surgery Registry
**North and South America**	
US	Intelligent Research in Sight (IRIS) Registry (supported by the American Academy of Ophthalmology) Medicare Database Paediatric Eye Disease Investigator Group (PEDIG) database Toddler Aphakia and Pseudophakia Treatment Study Registry
**UK and Europe**	
Denmark	Paediatric Cataract Register (PECARE)
Europe	EUREQUO (supported by the European Society of Cataract & Refractive Surgeons; ESCRS)
Germany	Germany Cataract Registry
Sweden	Swedish National Cataract Register
UK	National Ophthalmology Database (NOD) [supported by the Royal College of Ophthalmologists (RCOPhth)]

One of the most well-known examples is the Swedish National Cataract Register, which is the oldest nationwide cataract registry established in 1992 (Lundström et al., 2002). So far, it has produced >60 publications in the literature, covering many aspects of cataract surgery such as visual and refractive outcomes, posterior capsular rupture, endophthalmitis, postoperative practice pattern, and development of a composite risk-stratification scoring system, amongst others (Farhoudi et al., 2018; Zetterberg et al., 2021; Friling et al., 2022; Ridderskär et al., 2022). The European Registry of Quality Outcome for Cataract and Refractive Surgery (EUREQUO), which is supported by the ESCRS, represents another large-scale database that has so far captured more than 3 million cases of cataract surgery in Europe.^6^ This database provides pertinent surgical outcomes as well as the patient-reported outcomes following cataract and refractive surgeries, allowing the operating surgeons to audit their results and implement changes to their surgery (if required) to further improve the clinical outcomes. In addition, the big data obtained from this database (which included >2 million cases) has enabled effective analysis of risk factors for PCR and dropped nucleus during cataract surgery (Lundström et al., 2020; Segers et al., 2022).

In the UK, the Royal College of Ophthalmologists (RCOphth), UK, established the DHR-based National Ophthalmology Databases (NOD) in 2009, with an aim to monitor and improve the outcomes in cataract surgery and various ophthalmic conditions, including diabetic eye disease, age-related macular degeneration, glaucoma, and retinal detachment.^7^ In 2020, the NOD published the annual report on cataract surgery, which included >200,000 cataract surgery performed by >2,000 surgeons (web). The report highlighted that 86% of the eyes achieved at least 1 Snellen-line improvement in vision postoperatively. The overall PCR rate was 1.1%, with a higher rate (2.4%) in less experienced trainee surgeons and lower rate (0.9%) in consultant surgeons. Within the UK, all ophthalmic surgeons, including consultants and trainees, are required to record the number of cataract surgery performed and the rate of complications (particularly the rate of PCR), The findings from the NOD not only set important benchmarks for all the UK cataract surgeons but also help identify surgeons and trainees who require additional support and training on cataract surgery, particularly if the PCR rate is considerably higher than the national benchmark. These data have also been utilized to stratify the risk of PCR and vitreous loss, enabling the development of effective risk-stratification system to optimize patient selection and safety (Narendran et al., 2009; Day et al., 2015).

As mentioned above, IRIS Registry has showcased its clinical and research values in many fields of ophthalmology. Within the context of cataract surgery, Pershing et al. (2020) demonstrated a 0.04% rate of postoperative endophthalmitis among >8 million cases of cataract surgery and identified important predictive factors such as younger age, need for anterior vitrectomy, and when cataract surgery is combined with other ophthalmic surgeries. In addition, researchers were also able to utilize the IRIS Registry in analyzing and comparing the refractive outcomes and risk of endophthalmitis between immediate sequential and delayed sequential bilateral cataract surgery, which helps inform the clinical practice (Owen et al., 2021; Lacy et al., 2022).

In addition, the recent COVID-19 pandemic has caused an unprecedented surge in the service backlog and number of cases on the waiting list, particularly for cataract surgery (Ting et al., 2020b). With the availability of big data obtained from the EHR, it enables a comprehensive and systematic analysis of the utilization of the clinical and theater space, workflow efficiency (e.g., turnaround time between each cataract surgery), and supply-and-demand matching in terms of available workforce/resources and service backlog, which are useful for strategic planning and allocation of the resources within healthcare services. Big data from large-scale population-based studies also provide invaluable information on the service coverage and health equity (or inequity). For instance, effective cataract surgical coverage (eCSC) is often used as a measure to evaluate the service access to cataract surgery and the outcome of the surgery. A recent population-based study, based on 148 Rapid Assessment of Avoidable Blindness (RAAB) survey data from 55 countries involving ~500,000 adults aged 50 years and older, reported that eCSC varied considerably between countries, with higher rate in countries with greater income level, highlighting the need for increased efforts to improve access and quality of the surgery in under-resourced countries (McCormick et al., 2022).

## 4. Future directions

### 4.1. Integration of big data and artificial intelligence

The relationship between big data and AI-assisted technologies is highly synergistic and inextricably linked. The enormity and nature of big data usually require advanced computing power, software and algorithms (e.g., machine learning and deep learning-based AI algorithms) to process and analyze the data. On the other hand, development of highly accurate and generalizable AI algorithms often requires the input of big data that satisfy the attributed of “5 Vs”. With the rapid development of big data research and digital technologies in the recent years, it is anticipated that AI-power big data analytic platforms, coupled with telemedicine, will shape the future landscape of medicine (Sim et al., 2016; Ting et al., 2019a; Wu et al., 2019). The need for these innovative digital technologies in clinical practice was further heightened by the recent COVID-19 pandemic where all branches of healthcare services, including ophthalmology, have been severely impacted (Babu et al., 2020; Ting et al., 2020b,c, 2021f; Whitelaw et al., 2020; Ho et al., 2021; Kim et al., 2021).

So far, big data-driven AI technologies have demonstrated its clinical potential in many areas of corneal diseases and cataract. These encompass screening and diagnosing a wide array of conditions (e.g., keratoconus, IK, corneal opacity) and cataract, and preoperative planning for refractive surgery, to making automated clinical decisions for various diseases (Rampat et al., 2021). Studies have shown that the diagnostic accuracy of several AI algorithms can be as high as 92–97% in detecting keratoconus and preclinical keratoconus or forme fruste keratoconus (Arbelaez et al., 2012; Smadja et al., 2013; Hidalgo et al., 2016; Issarti et al., 2019; Lavric and Valentin, 2019; Ting et al., 2021b). Automated assessment of the corneal endothelial cell density in normal and diseased eyes as well as corneal guttata, based on AI-assisted algorithms using specular microscopy images and/or retroillumination slit-lamp photographs, have been developed to improve the management and follow-up in patients with corneal endothelial diseases and post-endothelial keratoplasty (Joseph et al., 2020; Vigueras-Guillén et al., 2020; Shilpashree et al., 2021; Soh et al., 2021; Karmakar et al., 2022). A recent study also reported the potential of machine learning algorithms in predicting the 10-year graft survival of PK and DSAEK using random survival forests analysis with highest variable importance factors (Ang et al., 2022). Understanding of the predictive factors allows the clinicians to address any modifiable preoperative factors, select the most appropriate type of keratoplasty for each individual patient, and optimize the long-term graft survival. This will also help reduce the need for regrafting, which has been shown as one of the most common indications for keratoplasty (Ting et al., 2012; Aboshiha et al., 2018).

In addition, several studies have demonstrated the ability of AI algorithms in diagnosing and differentiating the types of IK, and differentiating active IK from healed corneal scar (Liu et al., 2020; Lv et al., 2020; Koyama et al., 2021; Tiwari et al., 2022). These technologies are particularly useful in regions where resources and expertise are lacking. More recently, Li et al. (2020) reported the superior performance of a DL-based AI algorithm in diagnosing a wide range of corneal and conjunctival diseases, including IK, pterygium and conjunctivitis, and cataract based on using slit-lamp photographs. More importantly, the algorithm was able to provide automated clinical recommendation for further management, including clinical observation, medical treatment and surgical interventions. This can greatly reduce the diagnostic and referral time and improve the workflow efficiency within the healthcare system. For acute ocular conditions such as IK, timely diagnosis is crucial in achieving a good outcome; hence an effective AI-powered platform may serve as a novel diagnostic solution, particularly in LMICs.

Automated detection and grading of cataract as well as diagnosis of posterior capsular opacification by AI algorithms have also been described (Xu et al., 2020; Gutierrez et al., 2022). Furthermore, newer generations of IOL calculation formulae, based on big data-powered AI algorithms, have also been developed to enhance the predict accuracy of the IOL power (Ladas et al., 2021; Gutierrez et al., 2022). By using a dataset of ~5,000 patients, Li T. et al. (2022) recently demonstrated the ability of AI in enhancing the prediction of effective lens position and improving the accuracy of existing IOL formulas, including Haigis, Hoffer Q, Holladay, and SRK/T. Therefore, it is anticipated that the millions of data housed within several national cataract registries can be utilized to train and develop effect AI-based IOL calculation formulae and optimize the visual and refractive outcomes of cataract surgery in the near future.

### 4.2. Empowerment of big data research by internet of things, mHeath, and wearable devices

The potential of big data research has further been fueled by the recent rapid expansion and development in Internet of Things (IoT) technologies, mobile health (mHealth; a branch of medical and public health practice powered by mobile devices), and wearable devices (Kelly et al., 2020). So far, it has been estimated that more than 2 billion people own a mobile phone globally, with >50 million people utilizing app-based, interactive health self-care and self-triage (Millenson et al., 2018). The scopes and applications of mHealth range considerably from delivering health education, digital therapy, supporting clinical decision making for diagnosis and treatment, to improving clinical outcomes *via* behavioral modification (Rowland et al., 2020). Within the field of cornea, Inomata et al. (2020b) utilized crowdsourced big data, obtained from a mobile app (DryEyeRhythm) of around 3,000 participants, to identify participants with diagnosed and undiagnosed symptomatic DED and determine their associated risk factors. In addition, it was found that depressive symptoms are more common in individuals with DED, enabling an earlier detection and intervention for depression in this cohort of individuals (Inomata et al., 2020a).

Recent advances in wearable devices have also enabled real-time collection of millions of health datapoints (e.g., heart rate, blood pulse, step count, daily activity, etc.) for non-invasive diagnosis and monitoring of various diseases, including cardiovascular diseases, pulmonary diseases, hypertension, and diabetes, amongst others (Guk et al., 2019; Lu et al., 2020). Studies have shown that smart contact lenses (CLs) with biosensing technology are able to detect tear content and metabolites, including glucose and exosomes, which enable real-time non-invasive detection and monitoring of diabetes and cancer (Park et al., 2018; Li S. et al., 2022). The big data obtained from these biosensing technologies may be useful for CL wearers in the future where these smart CLs may help detect any early changes in the tear metabolites and inflammatory cytokines, which may help predict the risk of development of CL-induced DED, inflammatory and infectious keratitis.

In addition, Chen et al. (2021) recently developed a blink-sensing glasses to detect the blinking pattern between DED subjects and health controls. It has been recognized in the recent years that increased digital screen use (either for occupational, recreational or educational purpose) is a significant predisposing risk factor for DED (Mehra and Galor, 2020). Several mechanisms have been proposed, including reduced/abnormal blinking (which can lead to increased tear evaporation and ocular surface inflammation) and damage from the emitting light from the digital screen devices. Therefore, the big data generated from these blink-sensing glasses have the potential of monitoring the blinking patterns and behaviors of the digital screen users (or individuals with DED), which allows for an effective modification of the lifestyle and improves the management of DED.

### 4.3. Role of big data in predictive, preventive, personalized, and precision (P4) medicine

With the exponential increase in the availability of multi-omics data, large-scale population-based studies, EHR, and digital technologies, it is becoming possible to harness the power of these big data for implementing predictive, preventive, personalized, and participatory (P4) medicine. Instead of treating the patients reactively based on the presenting symptoms and signs, P4 medicine advocates personalizing the care to each individual at an individual molecular, cellular and organ levels, making the treatment more effective and cost-effective (Flores et al., 2013). Furthermore, the combination of real-time health data collected from these wearable smart devices with clinical and “multi-omics” data can potentially improve the understanding and management of certain multifactorial diseases (for instance, DED) where lifestyle and environmental factors play a vital role in the pathogenesis and phenotypic features of the disease (Inomata et al., 2020c).

Recently, Inomata et al. (2021) highlighted the potential of mHealth by using a data-driven multidimensional smartphone-based digital phenotyping strategy to assess and classify DED, which is a highly heterogeneous and multifactorial disease. A wide range of data, including demographics, medical history, lifestyle questions, blink sensing (*via* smartphone cameras and CIFaceFeature for facial detection), and daily subjective symptoms [using the Japanese version of the Ocular Surface Disease Index (J-OSDI)], were collected through the DryEyeRhythm mobile app. Subsequently, through hierarchical clustering heatmap, the authors were able to visualize and classify DED patients into several categories with distinct DED characteristics, illustrating the potential of P4 medicine in managing DED.

The potential of big data in shaping P4 medicine in ophthalmology is huge. For instance, with the increased availability of big data, it will be possible to personalize the choice of IOL for patients who are undergoing cataract surgery and predict those who are most likely to benefit from a certain type of IOL implant (e.g., multifocal vs. monofocal) in the future. One may also leverage the power of big data (and AI) to predict of the risk of postoperative complications following cataract surgery, which helps distinguish the group of patients who are suitable for community follow-up vs. hospital follow-up, thereby relieving the burden on the over-stretched healthcare services. Furthermore, with the combination of phenotypic and genotypic data as well as lifestyle factors (e.g., tendency for eye rubbing), it may be possible to predict the group of patients with keratoconus who are most likely to progress, thereby enabling early prophylactic intervention such as corneal cross-linking to prevent progression and maintain good vision.

### 4.4. Potential barriers for enabling big data research and its clinical potential

The potential values of big data, EHR, telehealth and mHealth in healthcare have long been recognized by the WHO, which was published as a report by the Global Observatory for eHealth in 2016 (World Health Organization, 2016). However, several barriers exist for translating their potential to real clinical values (Hulsen et al., 2019; Uslu and Stausberg, 2021). One of the main barriers is cost. Establishment and maintenance of these large-scale platforms, registries and EHR often requires substantial financial resources and workforce, which explains the scarcity of big data in under-resourced LMICs. Depending on the scale of the registries and EHR, they can cost the healthcare service from tens of thousands to multimillion dollars to set up and maintain the platforms within the healthcare services (Menachemi and Collum, 2011). Processing and analysis of big data also necessitates advanced computing power, facilities and expertise in order to arrive at clinically meaningful findings and conclusions. Furthermore, considerable expertise, workforce, resources, and facilities, all of which are associated with a high cost, need to be in place to prevent or reduce the risk of EHR system failure, which can significantly disrupt and paralyze the delivery of the healthcare services and cause harm to patients.

In addition to the cost and workforce, there remains a number of significant challenges associated with big data research, including data completeness, accuracy, heterogeneous data sources/platforms, data security, sharing, and visualization (Househ et al., 2017; Dash et al., 2019). Most large-scale EHR systems are designed for delivery of clinical services but not for evidence generation; therefore, capitalization of the EHR-derived big data for clinical research purposes is considerably challenged by the consistency, accuracy, and completeness of data. In addition, many of the systems are usually created for general medical and surgical services, which leads to inaccurate or incomplete data collection for ophthalmic diseases. For instance, many of the ophthalmic diagnoses/codes are not available in the general EHR systems, inhibiting an accurate assessment and analysis of the incidence, characteristics, causes, and impact of ophthalmic diseases presenting to the health services.

Another potential barrier for big data research is data privacy and sharing. Although there has been an increased availability of big data captured through various sources (Figure 1), processing of these data for research purposes are prohibited, unless: (1) the data are fully anonymized; (2) the data owner (i.e., the patients, healthy volunteers, etc.) provide “explicit consent”; or (3) the processing of data are necessary for provision of healthcare services or for public interest (Hulsen et al., 2019). In 2018, the European Union introduced a new set of regulations—the General Data Protection Regulation (GDPR)—to safeguard data privacy.^8^ It also places constraints on data sharing where appropriate consent needs to be obtained from the patient before the data can be shared with another organization. In addition, data sharing and management is further guided by the FAIR principles, which include “Findability,” “Accessibility,” “Interoperability,” and “Reusability” (Wilkinson et al., 2016). Therefore, to realize the potential of big data research in ophthalmology, all these highlighted barriers will need to be fully considered and addressed.

## 5. Conclusions

The continuous growth of IoT technologies, increased acceptability of mHealth, accessibility and affordability of mobile and wearable smart devices, and advancement in AI technologies in the coming years are likely to further expand the potential and applications of big data research in medicine and surgery, including ophthalmology (Wang et al., 2020; Rono et al., 2021; Dow et al., 2022). The establishment of big data resources such as corneal transplant registries, genomic studies, biobanks, and large scale EHR-based registries has so far provided a vast amount of valuable clinical and research information on cataract and a wide range of corneal diseases, ranging from non-sight threatening but functionally debilitating (e.g., DED) to sight threatening conditions (e.g., IK, PBK, keratoconus, etc.). Big data has advanced the understanding of many diseases, provided important benchmark for treatment and surgery, improved treatment outcome, and informed public policies. It is also anticipated that big data research will help propel the field of P4 medicine. However, there is currently a significant deficit and mismatch in the availability and demand for big data in LMICs, highlighting the need for increased effort and work to be invested in the under-resourced countries where blindness secondary to corneal opacity and cataract predominates (Pineda, 2015; Tan et al., 2015).

## Author contributions

Study conceptualization and design: DSJT and MA. Data collection and drafting of initial manuscript: DSJT. Critical revision of manuscript: RD, DSWT, and MA. All authors contributed to the data interpretation, analysis, and final approval of manuscript.

## Footnotes

^1^ https://www.ibm.com/blogs/watson-health/the-5-vs-of-big-data/

^2^ https://www.who.int/news-room/fact-sheets/detail/blindness-and-visual-impairment

^3^ https://restoresight.org/

^4^ https://www.odt.nhs.uk/statistics-and-reports/

^5^ https://www.ukbiobank.ac.uk/

^6^ https://www.escrs.org/about-escrs/registries/eurequo/

^7^ https://www.nodaudit.org.uk/resources/publications-annual-report

^8^ https://gdpr.eu/tag/gdpr/
